# Advances in HDL: Much More than Lipid Transporters

**DOI:** 10.3390/ijms21030732

**Published:** 2020-01-22

**Authors:** Soumaya Ben-Aicha, Lina Badimon, Gemma Vilahur

**Affiliations:** 1 Cardiovascular ICCC Program, Research Institute-Hospital de la Santa Creu i Sant Pau, IIB-Sant Pau, 08001 Barcelona, Spain; sbenaicha@santpau.cat (S.B.-A.); lbadimon@santpau.cat (L.B.); 2 School of Medicine, University of Barcelona (UB), 08001 Barcelona, Spain; 3 Centro de Investigación Biomédica en Red Cardiovascular (CIBERCV) Instituto de Salud Carlos III, 28001 Madrid, Spain; 4 Cardiovascular Research Chair, Universidad Autónoma Barcelona (UAB), 08001 Barcelona, Spain

**Keywords:** HDL, cardiovascular risk factors, cardiovascular disease, engineered HDL-mimetics

## Abstract

High Density Lipoprotein (HDL) particles, beyond serving as lipid transporters and playing a key role in reverse cholesterol transport, carry a highly variable number of proteins, micro-RNAs, vitamins, and hormones, which endow them with the ability to mediate a plethora of cellular and molecular mechanisms that promote cardiovascular health. It is becoming increasingly evident, however, that the presence of cardiovascular risk factors and co-morbidities alters HDLs cargo and protective functions. This concept has led to the notion that metrics other than HDL-cholesterol levels, such as HDL functionality and composition, may better capture HDL cardiovascular protection. On the other hand, the potential of HDL as natural delivery carriers has also fostered the design of engineered HDL-mimetics aiming to improve HDL efficacy or as drug-delivery agents with therapeutic potential. In this paper, we first provide an overview of the molecules known to be transported by HDL particles and mainly discuss their functions in the cardiovascular system. Second, we describe the impact of cardiovascular risk factors and co-morbidities on HDL remodeling. Finally, we review the currently developed HDL-based approaches.

## 1. Introduction 

**Figure 1:**
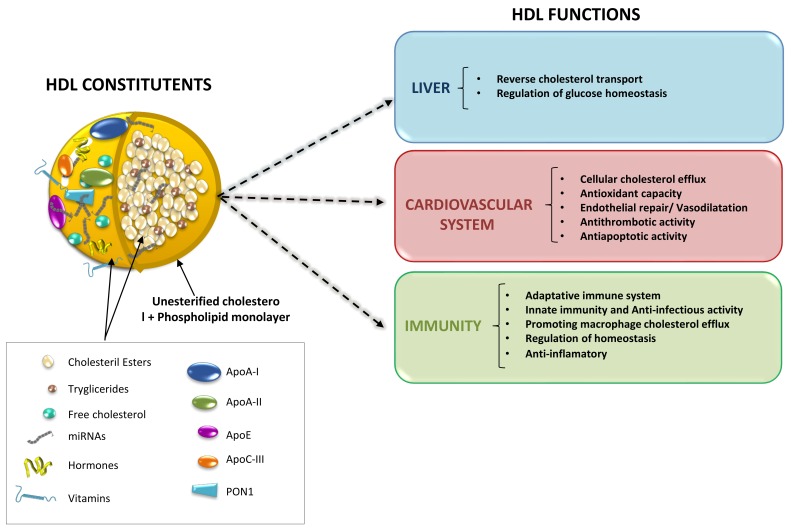
HDL main constituents and HDL-related functions in different cellular systems.

**Figure 2:**
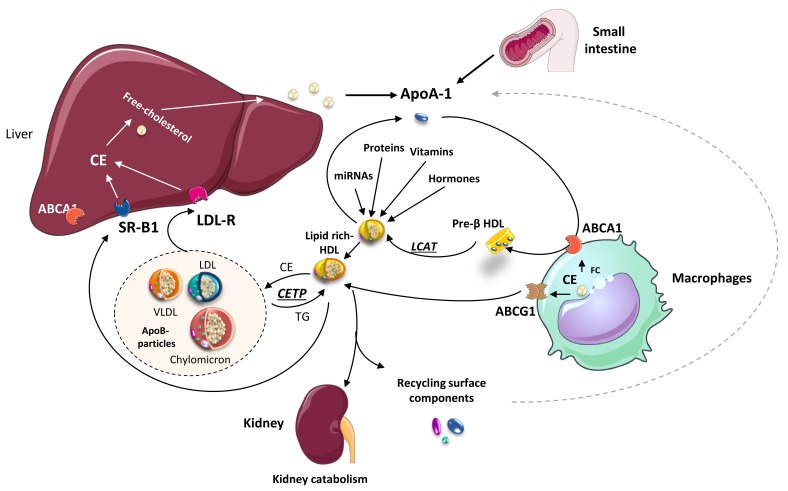
HDL biogenesis and its role in lipid metabolism. Nascent HDL particles (Pre-β HDL), formed from Apo-I secreted either by the liver or the thin intestine, uptake cholesterol from different cell types, including macrophages (via ATP binding cassette transporter A1; ABCA1). Concomitantly, other molecular constituents, including miRNAs, proteins, hormones, and vitamins, become incorporated into HDL-particles. Free cholesterol within the HDL is further transformed into cholesterol esters (CE) by the enzyme Lecithin: Cholesterol Acyltransferase (LCAT). Lipid-rich (mature) HDL particles exchange lipids with ApoB-containing particles [(chylomicrons, very-low-density lipoproteins (VLDL), low-density lipoproteins (LDL)] through cholesteryl ester transfer protein (CETP) enzyme and with macrophages via ABCG1. Finally, the liver receptors scavenger receptor (SR)-B1 and LDL-R transfer HDL lipids to the liver, ApoA1 is catabolized in the kidneys, and the remaining surface constituents are recycled.

High-density lipoproteins (HDL) represent a variety of lipoproteins with mean size of 8–10 nm and density of 1.063–1.21 g/mL [1]. The fundamental structure of an HDL particle includes a central core of esterified cholesterol, surrounded by a monolayer of phospholipids, free cholesterol, and apolipoproteins (apo), predominantly apoA-I and apoA-II (Figure 1) [2,3]. HDL size spans the spectrum from small, lipid depleted, discoidal particles, to larger, cholesterol-rich, spherical particles. The biogenesis of HDL occurs in the liver and intestine, which both synthesize and secrete apoA-I. Shortly after secretion as a lipid-poor protein, apoA-I interacts with the cholesterol–phospholipid transporter ABCA1 (ATP Binding Cassette A1) expressed by many cell types (including hepatocytes, enterocytes, and macrophages) to exchange lipids generating a nascent HDL particle (pre-β-HDL) [4]. The enzyme lecithin cholesteryl acyltransferase (LCAT) present on HDL particles favors the generation of cholesteryl ester, which forms the core of mature HDL particles [5]. Conversely, there are two metabolic pathways involved in HDL-cholesteryl ester clearance, which include a direct uptake by the liver or by steroidogenic tissues via the scavenger receptor BI (SR-BI); and, the transfer to apoB-containing lipoproteins (usually in exchange for triglyceride) by the plasma protein cholesteryl ester transfer protein (CETP). Uptake via SR-BI is selective, and after the removal of cholesteryl ester, the smaller apoA-I containing HDL particle dissociates and recycles [6]. In contrast, CETP not only contributes to HDL cholesteryl esters depletion but favors HDL-triglyceride enrichment. Triglyceride-enriched HDL particles are, in turn, susceptible to lipolytic modification by hepatic and endothelial lipases. Upon change by these two enzymes, the consequent smaller HDL particle becomes more sensitive to undergo faster catabolism [7]. Finally, apoA-I is mostly catabolized in the kidneys via a process involving the proteins cubilin and megalin (Figure 2) [8]. In summary, HDL becomes the primary vehicle for the transport of cholesterol from peripheral cells to the liver for excretion and catabolism. Yet, during this complex metabolic process, molecules other than lipids (i.e., hormones, vitamins, proteins, and miRNAs) are known to be incorporated into HDL particles and transported to distal organs, likely contributing to maintaining cardiovascular health.

In the following paper, we will first provide an overview of the molecules known to be transported by HDL particles and discuss the functions they are considered to regulate, describe the impact of cardiovascular risk factors and co-morbidities on HDL remodeling, and eventually review the HDL-based approaches developed so far.

## 2. Molecules Carried by HDL Particles

The study of HDL-particle composition has become a matter of high interest, particularly in light of the disappointing clinical data for HDL-cholesterol (HDL-C), raising therapies in secondary prevention and the lack of association between HDL-C and the risk of cardiovascular events in coronary artery disease (CAD) patients [9,10,11]. HDLs are complex molecules that, beyond lipids, are known to transport proteins, hormones, vitamins, and miRNAs, which are delivered to target tissues/cells. Implementation of mass spectrometry-based proteomic studies has allowed identifying 85 proteins associated with HDL particles. These proteins have been mainly related to the following main biological processes or molecular functions: lipid metabolism and transport, hemostasis, immune response, metal binding, and vitamin transport [12]. The most frequently detected proteins are ApoA-I and ApoA-II. ApoA-I is synthesized in both the intestine and the liver and constitutes approximately 70% of HDL protein size. ApoA-II is only synthesized in the liver, represents approximately 20% of HDL protein, and is present on about two-thirds of HDL particles in humans [13,14]. Besides apoA -I and -II, HDLs express other apolipoproteins, which, in order of detection, include ApoC-III, ApoL1, ApoA-II, ApoE, ApoC-I, ApoC-II, and ApoM [15]. At a lipid level, more than 200 lipid species have been identified to constitute HDL particles [16]. These are generally classified as neutral hydrophobic lipids (cholesteryl esters and triglycerides present in the HDL core), and amphipathic lipids (free cholesterol or phospholipids mainly transported on the HDL surface) [13]. The HDL lipid composition is comprised of 35–50% of phospholipids, 5–10% of sphingolipids, 30–40% of cholesterol esters, 5–10% of free cholesterol, and 5–12% of triglyceride [16]. HDL also carries hormones that bind to the HDL-apolipoproteins, including thyroxine (T4), retinol, estradiol, pregnenolone, and dehydroepiandrosterone [17]. HDL transport of hormones and their intercellular transfer are crucial for the endocrine and reproduction systems, particularly during pregnancy [18,19]. Finally, HDL also incorporate vitamins, including carotenoids (lipophilic precursors for vitamin A) and vitamin E. As to the former, although HDLs transport a small percentage of nonpolar carotenoids (e.g., lycopene, 17%; α-carotene, 26%; and β-carotene, 22%), they carry a large proportion of polar carotenoids (lutein, 53%; cryptoxanthin 39%) [20,21]. Carotenoids are mainly delivered to the eye retina where they are accumulated in the macula, likely protecting against its degeneration, cataract development, and blindness. Moreover, carotenoid treatment has been associated with an increase in HDL reverse cholesterol transport capacity in mice [22] and has been shown to protect against early atherosclerosis development [23]. As per vitamin E, HDL has demonstrated to deliver this vitamin to epithelial cells, although its exact role remains uncertain [20].

Finally, within the last years, it has become evident that HDLs also transport miRNAs, short (~22 nucleotides), non-coding, single-stranded RNA molecules that regulate the expression of target genes by blocking its translation or promoting its degradation. So far, miR-33a, miR-30c, miR-92a, miR-122, miR-125a, miR-126, miR-145, miR-146a, miR-150, miR-155, miR-223, miR-378, miR-486, and miR-17/92 cluster have [24,25] been detected in HDL particles and have been suggested to regulate crucial pathways for maintaining cardiovascular homeostasis [26]. Particularly, miRNA-33a and miRNA-223 have been described to protect against atherosclerosis by modulating HDL cholesterol efflux capacity and lipid metabolism [27,28].

## 3. Protective Effects of HDL Particles beyond Lipid Removal 

Since the Framingham Heart Study in the 1960s–1980s [29], many epidemiological and experimental studies have supported a beneficial role of HDL particles in the cardiovascular system. The critical benefit attributed to HDL particles relies on their ability to induce reverse cholesterol transport (RCT) [30,31,32]. HDL loss of cholesterol efflux capacity is associated with cardiovascular diseases [33,34,35]. However, HDLs exert several other benefits that protect the cardiovascular system, including antioxidant, anti-inflammatory, vasodilatory, antithrombotic, immunomodulatory, and endothelial repair properties, including the recruitment of endothelial progenitor cells [3,36,37,38]. Many clinical data, however, support the fact that HDL particles isolated from CVD patients have their benefits altered, including lower cholesterol efflux capacity and less anti-oxidant and anti-inflammatory properties, compared with healthy subjects [39,40,41,42]. Furthermore, HDL particles have been reported to even be deleterious [43,44]. As such, HDL isolated from CAD patients and antiphospholipid syndrome have been shown to promote apoptosis by stimulating p38-mitogen-activated protein kinase-mediated activation of the proapoptotic Bcl-2 protein tBid and to reduce nitric oxide (NO) bioavailability, impairing HDL anti-inflammatory and antioxidant properties, respectively.

### 3.1. Antioxidant and Anti-Inflammatory Effects

HDLs have experimentally been shown to protect against LDL oxidation, thereby preventing the generation of proinflammatory oxidized lipids, mainly lipid hydroperoxides and short-chain oxidized phospholipids [36,45,46,47]. The main antioxidant enzyme carried by HDL particles is paraoxonase 1 (PON1). PON1 is a member of a family of proteins that also includes PON2 and PON3. PON1 and PON3 are part of the HDL particles, while PON2 is found in a wide variety of tissues, such as endothelial cells, smooth muscle cells, and macrophages [48]. Interestingly, besides its antioxidant potential, PON1 has also been suggested to play a crucial role in HDL-endothelial nitric oxide synthase (eNOS) activation. PON1-deficient mice have failed to stimulate endothelial nitric oxide (NO) production, leading to impaired endothelium protection and repair [37]. HDL has also demonstrated, however, to activate eNOS through an SR-B1-associated intracellular kinase cascade activation [49]. 

Other mechanisms by which HDL may modulate inflammation partly involve miR-223. In this regard, in vitro studies have demonstrated that macrophages (J774) can transfer miR-223 to HDL [26], which, in turn, exert anti-inflammatory effects through HDL-miR-223 delivery and translational repression of intercellular adhesion molecule (ICAM)-1 in endothelial cells [50].

### 3.2. Protection against Ischemia/Reperfusion

Ischaemic heart disease, mainly presented as myocardial infarction (MI) due to occlusion of an epicardial coronary artery, remains the leading cause of death and disability worldwide. If timely reperfusion is not achieved and no collateral circulation is present, cardiomyocytes will be irreversibly damaged and die and substituted by fibrous scar tissue with impairment of myocardial contractile function [51]. However, reperfusion, although mandatory to salvage ischemic myocardium from cell death, contributes per se to enhance cardiac injury by affecting myocytes not yet severely damaged by the ischemic insult, the so-called ischemia/reperfusion (IR) injury [52]. Theilmeier et al. were the first to demonstrate in an in vivo mouse model of MI that HDL markedly reduces the size of infarction [53]. Previous findings further demonstrated, in a translational animal model with clinical impact and by magnetic resonance imaging analysis, that HDL infusion not only limited cardiac damage, but also improved cardiac contractility as compared to infusion with vehicle [54,55,56]. Besides HDL particles, its component sphingosine-1-phosphate (S1P), which is mainly bound to ApoM, has also been shown to protect against IR injury [57,58], and administration of fingolimod, an S1P receptor agonist, has demonstrated to limit the size of infarction and improve cardiac performance in a pig model of MI [59].

miRNAs transported by HDL have also been shown to protect against IR. For instance, reduction of miR-92a levels has been shown to reduce infarct size and post-ischemic loos of cardiac function by displaying cell-protective, pro-angiogenic, and anti-inflammatory effects, whereas miR-486 has demonstrated to prevent cardiomyocyte apoptosis execution [60,61,62,63]. These promising experimental data have encouraged the development of synthetic HDL-like formulations containing, within the structural HDL components, locked nucleic acid-modified antisense against a given miRNA or different proportion of these candidate miRNAs [64].

### 3.3. Endothelial Progenitor Cell Recruitment

Considerable experimental evidence supports the ability of HDL particles to display a plethora of beneficial effects on the endothelium [32,46,65]. Besides inducing eNOS activation and NO production, HDL has been shown to inhibit endothelial expression of adhesion molecules, reduce apoptosis execution, and promote EPCs-mediated endothelial cell repair [39]. Particularly, ApoA-I has been shown to enhance re-endothelialization post-percutaneous coronary intervention and post-stent aortic implantation in ApoA-I infused animals [66,67,68] by enhancing EPCs recruitment, reducing neointimal hyperplasia, and inhibiting the inflammatory response [69]. Moreover, engineered-HDL formulations have also been demonstrated to increase EPC mobilization in diabetic patients [70]. Although the mechanisms behind EPC mobilization remain poorly understood, cell culture studies have suggested that it involves HDL-SR-BI interaction and [49,71] activation of the CD36- mitogen-activated protein kinase (MAPK)-thrombospondin-1 pathway [72].

### 3.4. Antithrombotic Effects

HDL antithrombotic properties have been reported within both the arterial and venous beds [3,73]. The ability of HDLs to lessen the thrombotic risk is thought to be mediated by several mechanisms involving a reduction in platelet susceptibility to aggregation and attenuation of the activation of the coagulation cascade. HDL has been shown to increase endothelial NO and prostacyclin synthesis and bioavailability [74], thereby preventing platelet activation from occurring, to attenuate vascular expression of tissue factor and exert anticoagulant cofactor activity by enhancing activated protein C (APC): protein S. This leads to a diminishment in thrombin generation, a potent platelet inducer, and precursor of fibrin formation [49].

### 3.5. Immunomodulatory Properties

Several clinical trials have supported a link between HDL and immunity in the setting of autoimmune diseases (e.g., systemic lupus erythematosus ankylosing spondylitis, and rheumatoid arthritis) [75,76]; yet, the immunomodulatory properties of HDL within the cardiovascular system remains far less understood. Accumulating data have suggested that HDLs are involved in host defense as part of both the innate and adaptive immune system [38,77,78]. In this latter regard, HDL has demonstrated to affect T cell activation and signaling by altering cholesterol content within the lipid rafts [79,80]. Furthermore, HDL has also been shown to inhibit the ability of antigen-presenting cells (APCs) to stimulate T cells, regulate complement activation, and block the conversion of monocytes into migratory dendritic cells (DCs) through the HDL-associated platelet-activating factor-acetylhydrolase [81]. 

## 4. Impact of Cardiovascular Risk Factor and Co-Morbidities on HDL Composition and Function 

Accumulating evidence suggests that HDL function and composition are found to be altered in the presence of overt cardiovascular disease. As such, the presence of CAD, MI [82], heart failure [83], and stroke [84] have been associated with impaired HDL anti-atherogenic properties (i.e., stimulation of vascular NO production, anti-oxidative and anti-apoptotic potential, and cholesterol efflux capacity) [43,85]. In addition, we cannot forget that clinical trials (particularly phase III) include elderly patients under multiple drug treatments. In this regard, the use of statins has been suggested to abolish HDL-related benefits. [86]. Moreover, the presence of cardiovascular risk factors, including hypercholesterolemia [13,60,87], obesity [88], diabetes type 1 and 2 [89,90], and even frequent exposure to pollution [91,92,93] or ethnicity have demonstrated to impact HDL properties and induce structural changes leading to variations in their subtypes profile. Altogether, these conditions may help explain the disappointing results observed in clinical trials aiming to increase HDL-c levels in secondary prevention. The venue of new technology systems, like genome-wide association studies (GWAS), has allowed assessing genetic predisposition to display improved or diminished HDL-related benefits. For instance, recent studies have reported that the cholesterol efflux capacity is a heritable trait and that certain genetic polymorphism in genes involved in HDL and triglycerides metabolism are associated with less HDLs RCT and anti-oxidative capacity [94,95,96]. We have recently demonstrated that HDL remodeling is affected by low-density lipoprotein (LDL)-cholesterol levels. As such, we have shown in pigs that the presence of hypercholesterolemia modifies HDL lipidomic and proteomic profile and renders HDL particles dysfunctional [56]. Particularly, HDL particles formed under hypercholesterolemic conditions, as compared to HDL particles formed in a normocholesterolemic milieu, have lower levels of phosphatidylcholine-lipid species and higher levels of cholesteryl esters (i.e., mature HDL particles with lower membrane fluidity) and a lower cargo of several proteins with known cardioprotective functions, including ApoM and lipocalin retinol-binding protein-4. Interestingly, such adverse structural remodeling is associated with a marked reduction in HDL antioxidant and cholesterol efflux capacity and complete loss of HDL-related cardioprotection [13].

At an epigenetic level, the presence of co-morbidities has also been shown to induce changes to the HDL-miRNA signature [60]. As such, an association between HDL-miRNAs profile and CAD stability has been established [97], and the content of HDL-miR-223 is found to be altered in the presence of insulin resistance and familial hypercholesterolemia subjects [26,98,99,100,101,102]. Extending these previous findings, we have recently evidenced that hypercholesterolemic HDL particles transport higher levels of miR-126 as compared to native-HDL, which in turn is delivered to endothelial cells through an SRB1-dependent mechanism likely modulating HIF-1α expression [103]. 

Finally, HDL size, composition, and functionality are being studied as potential biomarkers to predict microangiopathy, coronary artery disease [104,105], aortic aneurysm [106], and atherosclerosis [107]. In this regard, we have detected alterations in ApoJ proteomic profile due to differential glycosylation patterns in acute MI-patients within the first six h of onset of the event, becoming a potential early marker of acute MI [108]. Noteworthy, changes in lifestyle behavior, like implementing exercise and a healthy diet daily, have demonstrated to restore and enhance HDL protective properties. As such, regular intake of Mediterranean diet-related functional foods, such as fermented beverages or tomato, have experimentally shown, not only to enhance HDL levels but, most importantly, to improve HDL functionality (i.e., antioxidant potential) [109,110,111,112].

## 5. HDL-Based Approaches

The venue of nanotechnology has provided a platform for the development of reconstituted HDL (rHDL) or synthetic HDL (sHDL). These HDL-mimicking particles are derived from cholesterol-free HDL particles (prepared from purified plasma or recombinantly expressed) and ApoA-I or short synthetic ApoA-I mimetic peptides [like ApoAI Milano (ApoA-IM)] complexed with phospholipids (Table 1). Of note, the number of ApoA-I chains incorporated into the particle, and its overall size is the primary mechanism by which HDL-mimetic particles are classified and labeled [113]. Such inorganic nanoparticles that mirror HDL composition provide a scaffold to create synthetic structures that mimic mature, spherical, or discoidal forms of HDL. The resultant nanoparticle retains both the physicochemical properties as well as the biological functions of the native HDL. As such, these “empty” particles have demonstrated to be highly effective in cholesterol efflux from lipid-laden cells both in vitro and in vivo and to target SR-BI-expressing cells [114,115]. These engineered nanoparticles can act as versatile drug delivery carriers of miRNAs, specific proteins, and drug-delivery systems. As such, the ability of rHDL/sHDL to deplete cellular cholesterol and target SR-BI along with the biocompatibility of the individual components and proven clinical safety has enhanced the interest of developing rHDL/sHDL constructs in different medical areas, such as cancer, where HDL-like particles are being used for anti-cancer therapeutics and tumor imaging chemotherapy [116].

### 5.1. HDL-Mimetics: Composition and Characteristics

Despite all these advances, there are specific nanoarchitecture requirements that cannot be overlooked [117,118]. One of the most important is to prolong HDLs’ mimetics life-span to retain the molecule on the organism long enough to enhance its beneficial properties and reduce the maintaining dosage. This feature is mainly determined by the type, proportion, and interaction between lipid and protein cargo [119]. Many of the early structural studies on rHDL/sHDL employed particles reconstituted with dimyristoylphosphatidylcholine (DMPC, a shorter saturated acyl chain PL, 14:0), due to its ability to spontaneously form rHDL/sHDL particles when DMPC vesicles were mixed with apoA-I [120,121,122,123,124,125]. Later, the cholate dialysis method [126] was reported to allow reconstitution of rHDL/sHDL with physiologically relevant phospholipids that have longer and unsaturated acyl chains like palmitoyloleoylphosphatidylcoline (POPC, 16:0–18:1). Schwendeman A et al. reported that rHDL-POPC displayed enhanced anti-inflammatory properties compared to rHDL rich in sphingomyelin, and consequently, had higher atheroprotective effects [127]. Overall, HDL formulations should aim to increase the proportion of protein and reduce the percentage of lipid since this approach confers HDL-mimetics with a longer lifespan and solid structure at the same time, retaining their flexibility enough for cell and tissue interactions. Moreover, HDL-engineered particles can then be further stabilized by protecting them from degradation with the conjugation of small RNAs (siRNAs or miRNAs) [128].

**Table 1:** HDL synthetic-based approaches.

HDL-Mimetic	Composition	Mechanism of Action	Experimental Design	Added Capacity (Compared to Native HDLs)	Reference
**ETC-216**	Human recombinant Apolipoprotein A-I Milano	ABCA1	Stopped ClinicalTrials.gov Identifier: NCT02678923	__	[129]
**MDCO-216**	Human recombinant Apolipoprotein A-I Milano	ABCA1	Phase 2 clinical trial: ClinicalTrials.gov Identifier: NCT02678923	Increases ABCA1-mediated cholesterol efflux and pre-beta 1 HDL.	[130]
**CSL112**	Human plasma-derived apolipoprotein A-I (apoA-I)	ABCA1	Phase 2a in stable atherothrombotic patients and Phase 2b for patients with acute MI: AEGIS-I trial ClinicalTrials.gov NCT02108262	Increases cholesterol efflux capacity	[131]
**rHDL-apoA-I**	Human plasma-derived apolipoprotein A-I (apoA-I)	ABCA1	Phase 2 on atherosclerosis ClinicalTrials.gov Identifier: NCT00225719	Increases cholesterol efflux capacity	[132,133]
**CER-001**	Lipoprotein complex mimicking discoidal pre-β HDL, consisting of recombinant human apoA-I	ABCA1	Phase 2 studies: CHI-SQUARE and CARAT trials clinicaltrials.gov Identifier: NCT01201837 and NCT02484378 respectively	Can rapidly mobilise large amounts of cholesterol into the HDL fraction	[134,135]
**Nanolipoprotein Particles (NLPs)**	Phospholipid bilayer stabilized by an apolipoprotein scaffold protein	ABCA1	Initial in vitro state	Enhanced particle stability	[114]
**sHDL-T1317**	sHDL apoA-I peptide+A synthetic LXR agonist, T0901317 (T1317)	ABCA1	Preclinical studies	Upregulates the expression of ATP-binding cassette transporters and increases cholesterol efflux in macrophages in vitro and in vivo.	[136]
**ApoE-Based rHDL**	rHDL particles containing ApoE3	ABCA1 and LCAT	Phase 1 in China, preclinical studies in Europe	Enhances endosomal/lysosomal escape capacity	[137,138,139]
**rHDL-DiR-BOA**	Mimicking peptide phospholipid scaffold (HPPS)	SR-B1	Initial in vitro state	Endosomal/lysosomal avoidance capacity which makes a highly biocompatible, exhibited long circulation half-life in serum nanocarrier	[140]
**cp-rHDL**	rHDL+cell penetrating peptides	__	Initial in vitro state	Easily overcome the cellular plasma membrane	[141,142]
**Receptor-Mediated rHDL Cellular Internalization**	rHDL+cell receptor signalling structures	SR-B1	Initial in vitro state	Enhances the accumulation of nanoparticles and increased uptaking	[143,144]
**rHDL-siRNA**	rHDL+siRNAs	SR-B1	Initial in vitro state	Allows directed siRNA delivery	[142]
**AT-DXS-LP-rHDL**	Atorvastatin calcium (AT)-loaded dextran sulfate (DXS)-coated core-shell reconstituted high-density lipoprotein (rHDL)	SR-AI	Initial in vitro state	High-affinity SR-AI as well as depletion of intracellular cholesterol by apoA-I mediated cholesterol efflux	[145]
**rHDL-AuNP**	AuNPs+rHDL+ApoE	Receptor-mediated endocytosis in glioblastoma cells	Initial in vitro state	A platform for transport and delivery of hydrophobic gold nanoparticles	[146]
**rHDL-rApoJ**	phospholipids with recombinant human ApoJ (rApoJ)	Amyloid beta (Aβ) interaction	Preclinical studies	Maintains the ability to prevent the Aβ fibrillation and mediated higher cholesterol efflux from cultured macrophages	[147]
**sHDL-EL**	sHDL+Substrate for plasma endothelial lipase (EL) with useful specificity	Endothelial lipase	Initial in vitro state	Specificity for EL	[148]

### 5.2. Potential Clinical Applicability of HDL Mimetics

So far, several “HDL mimetics” have been successfully tested in experimental animal models for their ability to reduce the burden of atherosclerotic cardiovascular disease [149,150]. In contrast, however, they have failed to continually prove beneficial effects in the clinical setting [151] (Table 1), and most importantly, none have demonstrated to reduce mortality, which likely reflects the complexity of the association between HDL and atherosclerotic cardiovascular disease. Moreover, it also suggests that HDL particles may undergo remodeling when infused to patients that suffer from many cardiovascular risk factors or are under drug treatment.

Serial infusion of the ApoA-I Milano phospholipid complex ETC-216 demonstrated in Phase I clinical trials to exert profound lipid changes to an extent, comparable to that found in ApoA-I Milano mutation carriers. Furthermore, the infusion of ETC-216 resulted in a reduction in atherosclerotic plaque burden [152] in line with our previous observations in atherosclerotic rabbits [153]. However, ETC-216 development was halted because of the presence of side effects, including a dose-dependent increase in neutrophil and a concomitant decline in lymphocyte levels, leading to multi-organ failure. Interestingly, a careful evaluation of the entire manufacturing process revealed that ETC-216 contained small quantities of residual host cell proteins that elicited an immune response. Consequent removal of the contaminant proteins from the nanoconstruct and essential improvements in the downstream manufacturing process led to the development of a recombinant ApoA-I Milano formulation named MDCO-216. MDCO-216 has been tested in healthy volunteers and patients with stable CAD, showing modulation of lipid parameters and an increase of cholesterol efflux with no adverse effects [129,130]. More recently, MDCO-216 has also reported, at an experimental level, to revert heart failure progression in Type 2 diabetic mice as well as restore cardiac function [154], lower myocardial acetyl-coenzyme A carboxylase levels, and decrease myocardial transforming growth factor-β1 [155]. CSL112, a reconstituted, infusible, plasma-derived ApoA-I structure, is another promising HDL-mimetic. CSL112 administration has been shown to enhance the quantity and functionality of plasma ApoA-I as well as promote ABCA1 dependent cholesterol efflux capacity, becoming an attractive option for rapid cholesterol plaque removal [131]. Finally, CER001 is a lipoprotein complex mimicking discoidal pre-β HDL consisting of recombinant human ApoA-I. Yet, so far, CER001 has demonstrated no benefits in reducing coronary atherosclerosis assessed by intravascular ultrasonography and by coronary angiography when compared with the administration of placebo [152].

## 6. Concluding Remarks

In summary, HDLs are complex particles constituted by a vast array of lipids, proteins, hormones, vitamins, and miRNAs, which confer HDL particles with multiple cardiovascular protective properties mainly related to atherosclerosis development. Advances in our outstanding on the structural components of HDL particles in different physiological and pathological conditions (HDL-remodeling) have allowed us to better understand the potential changes that may account for the loss of HDL function. Moreover, it has provided us with potential targets amenable for therapeutical modulation to restore HDL functionality or enhance specific protective features. In this regard, the implementation of new platforms aimed at designing and developing different reconstituted HDL formulations has offered a unique opportunity to overcome HDL-related structural alterations in the presence of co-morbid conditions or risk factors as well as to deliver drugs to target cells accurately. There is, however, much work that needs to be done in the arena of HDL-nano synthesis to obtain HDL constructs that remains stable and effective over time in the body and more closely resemble the healthy and native HDL particles endowed with multiple cardiovascular benefits (Table 1) [114].
